# Characterizing the Flowering Phenology of *Rosa rugosa* Thunb. as an Ecosystem Service in the Context of Climate Change in Kupinovo (Vojvodina), Serbia

**DOI:** 10.3390/plants14121875

**Published:** 2025-06-18

**Authors:** Mirjana Ljubojević, Jelena Čukanović, Sara Đorđević, Djurdja Petrov, Nevenka Galečić, Dejan Skočajić, Mirjana Ocokoljić

**Affiliations:** 1 Faculty of Agriculture, University of Novi Sad, Trg Dositeja Obradovića 8, 21000 Novi Sad, Serbia; mirjana.ljubojevic@polj.uns.ac.rs (M.L.); jelena.cukanovic@polj.uns.ac.rs (J.Č.); sara.djordjevic@polj.edu.rs (S.Đ.); 2 Faculty of Forestry, University of Belgrade, Kneza Viseslava 1, 11030 Belgrade, Serbia; nevenka.galecic@sfb.bg.ac.rs (N.G.); dejan.skocajic@sfb.bg.ac.rs (D.S.); mirjana.ocokoljic@sfb.bg.ac.rs (M.O.)

**Keywords:** shrub roses, flowering stages, reblooming, frost damage, landscape sustainability

## Abstract

Given the growing impact of climate change, this study examines the flowering phenology of *Rosa rugosa* Thunb. in Kupinovo (Vojvodina, Serbia). Data collected over 18 years (2007–2024) were analyzed to assess changes in primary flowering, while secondary flowering was monitored from 2022 to 2025. Phenological stages were recorded every other day, and dates were converted into day-of-year (DOY) values. Heat accumulation (GDD) was calculated using daily max/min temperatures and thresholds. In 2024, *R. rugosa* exhibited a 37-day earlier onset and a 50.4-day later completion of primary flowering compared to previous years. The variability of key phenological events of primary flowering was observed in the interaction with climatic parameters, with regular fruiting. The species proved tolerant to heat and drought, suggesting potential range expansion. Optimal temperatures for secondary flowering were identified: abundant flowering occurred at 13.6 °C max and 4.9 °C min, while moderate flowering occurred at 9.0 °C max and 4.2 °C min. Regression analysis confirmed the positive effect of rising temperatures on flowering intensity. While freezing halted secondary flowering and damaged open buds, unopened buds remained unaffected. These findings highlight *R. rugosa* as a resilient, ornamental species, relevant to climate adaptation strategies, nature-based solutions, and the preservation of ecosystem services under global warming scenarios.

## 1. Introduction

The concept of ecosystem services has been promoted internationally as a way to communicate and understand the relationship between people and nature [1]. It provides an opportunity to translate biophysical issues into socially relevant terms, facilitates communication with decision-makers [2], and enhances public engagement within various planning processes, including landscape planning and design [3]. In these processes, shrub plants have significant implications for ecosystem functioning and services [4], depending on climatic variables, soil characteristics, and specific shrub traits [5]. The presence of shrub plants provides benefits such as reducing soil surface temperature, increasing infiltration and water content [6], and enhancing soil nutrient levels [7]. However, it should be noted that ecosystems with a high percentage of shrub plants are dynamic, and changes in the relative abundance of grass and woody plant species can impact ecosystem functioning over decadal time spans [8]. Therefore, long-term studies are needed to assess the benefits of shrub plants in ecosystem functioning and services under climate change conditions.

In this regard, one of the shrub roses, which are also known as landscape roses [9,10], was selected as they are a significant indicator of meteorological and climatic conditions [9]. The genus *Rosa* L. is one of the most diverse genera of ornamental, mostly shrub plants, with a large number of cultivars [11,12], which have been cultivated for thousands of years for ornamental purposes in landscape design [13], but also for use in medicinal, cosmetic, and food applications [14]. Biodiversity and the diverse origins of roses lead to variations in their cultivation requirements and ornamental characteristics. Given this, and considering that the adaptability and sustainability of wild rose species in Vojvodina (Serbia) have not been thoroughly studied, particularly in the context of climate change, the focus of this research is *Rosa rugosa* Thunb. Rugosa Rose belongs to the most diverse subsection *Rosa* (Eurosa) in the section *Cinnamomeae* (DC.) Ser. 1825 [10,11]. Native to the coastal regions of the Far East (from the Korean Peninsula to Sakhalin), it was introduced to Europe around 1796 [11]. It is classified as an invasive neophyte in Europe, where it forms large dominant populations, especially in coastal areas. It is highly resistant to air pollution, drought, poor soils, and salinity [15]. On the other hand, it is also a species from the order of aromatic plants, valued for the extraction of essential oils, as well as for use in landscape planning and design [13].

However, trends in global warming are altering phenology, including phenophases such as flowering, as confirmed by many phenological studies [16,17,18,19]. Due to the interdisciplinary nature of phenological data, they are significant indicators for climate change, ecology, ecosystem processes, biodiversity, forestry, horticulture, landscape architecture, and human health and well-being [17,20]. In the case of ornamental plants, such as *R. rugosa*, which are introduced into the environment mainly for their visual values, with primarily flowering as a key element of their ornamental appeal [18,21] and the duration of flowering, detailed analyses are required for appropriate selection [13]. This is especially true for secondary flowering after primary flowering, which is known as repeated blooming for most “modern roses” [22]. Secondary flowering has high ornamental value, and its foundation lies in the influence of exogenous factors such as air temperature [23]. To enable a better understanding of climate change effects, phenological datasets on flowering must include multiple flowering phases and records on the duration of flowering. Moreover, the recording and interpretation of climate data should consider the developmental cycle underlying the phenological patterns of flowering [17,24]. Proper selection of plant material for diverse landscapes is of economic importance, as plant choices affect maintenance costs [19,21].

Accordingly, the aim of this study was to investigate the flowering phenology of *R. rugosa* in response to changing meteorological conditions in Kupinovo (Vojvodina), Serbia, based on nineteen years of observations. To examine the potential additional values for *R. rugosa* as an ornamental plant providing multiple ecosystem services, the specific objectives were (a) to characterize the range of air temperatures in which the species is found; (b) to investigate the phenological patterns of the primary and secondary flowering of the species in the context of changing maximum and minimum air temperatures; (c) to identify the maximum and minimum temperatures at which *R. rugosa* was damaged; and (d) to assess the suitability of *R. rugosa* as a landscape element providing multiple ecosystem services. Furthermore, the new research approach enables the optimization of growing conditions for the species based on predictions of acclimatization speed and can support the process of selecting the most suitable plants to achieve sustainable visual, economic, and ecological effects in the landscape.

## 2. Results

### 2.1. Weather Conditions

Based on the analysis of climatic parameters [25], noticeable trends include an increase in average, minimum and maximum air temperatures, a rise in the number of summer and tropical days, variations in precipitation sums, and an increase in the number of days with precipitation ≥1.0 mm, indicating extreme climatic events. Additionally, there is a variation in the monthly number of sunshine hours during the study period compared to the reference period (1991–2020), as well as deviations in these parameters in 2024 compared to both the reference period and the period 2007–2023 (Table 1).

For the flowering of *R. rugosa*, the mean seasonal air temperatures are important, and a trend of increasing temperatures is noticeable during the study period compared to the reference period. The mean seasonal maximum and minimum temperatures also show a linear growth trend. The largest deviation occurred in the winter season, when the mean temperature was higher by (a) 1.1 °C for maximum temperatures during the study period compared to the reference period, and 5.2 °C and 4.1 °C in 2024 compared to the reference period and the study period, respectively; and (b) 1.0 °C for minimum temperatures during the study period compared to the reference period, and 3.5 °C and 2.5 °C in 2024 compared to the reference period and the study period, respectively.

The next significant deviation occurred in the summer season, when the mean temperature was higher by (a) 0.7 °C for maximum temperatures during the study period compared to the reference period, and 3.5 °C and 2.8 °C in 2024 compared to the reference period and the study period, respectively; and (b) 0.7 °C for minimum temperatures during the study period compared to the reference period, and 3.1 °C and 2.3 °C in 2024 compared to the reference period and the study period, respectively.

The mean relative humidity of the air shows a linear downward trend, with the most pronounced decrease observed during the winter season, where lower values of 0.7% were recorded for the study period compared to the reference period. The number of sunshine hours across all seasons shows a linear upward trend.

The annual amount of precipitation in the research period (2007–2023) was higher by 2.5 mm compared to 1991–2020. During 2024, there was a significant surplus of precipitation in May, June, July, and September, and a deficit in April, August, and October, which is expressed as a deviation of the sum of precipitation (mm) for April −24.1 (compared to 1991–2020), that is −18.2 (compared to 2007–2023), May +31.7 (compared to 1991–2020), i.e., +17.7 (compared to 2007–2023), June +12.4 (compared to 1991–2020), i.e., +17.1 (compared to 2007–2023), July +11.2 (compared to 1991–2020) or +18.8 (in compared to 2007–2023), August −47.5 (compared to 1991–2020) or −39.7 (compared to 2007–2023), September +30.8 (compared to 1991–2020) or +34.8 (compared to 2007–2023) and October −6.7 (compared to 1991–2020), respectively, −4.1 (compared to 2007–2023). The increase in the number of days with precipitation ≥ 1.0mm by 5.4 in the period 2007–2023 compared to the reference period is highlighted, which indicates extreme climatic events. During 2024 there were 4 such days in April, 17 in May, 14 in June, 5 each in July and August, 14 in September, and none were recorded in October.

The results of the mean monthly air temperatures (°C) and precipitation totals (mm), categorized by percentiles and terciles, are presented in Table 2 and Table 3 for the period April–October 2024, during which the primary flowering of *R. rugosa* occurred consecutively for 18 years. In April, the air temperature was categorized as very warm according to the percentiles and warm according to the terciles, with precipitation falling into the drought category both by percentiles and terciles. In May, the air temperature was in the normal category for both percentiles and terciles, with precipitation in the rainy category. In June, July, and August, it was extremely warm, with variable precipitation ranging from the normal category through the rainy category to the extremely dry category in August. In September and October, the air temperature was classified as warm, with precipitation in the rainy category, and in October, precipitation was in the normal category.

### 2.2. Phenological Patterns of Primary Flowering

**Figure 1:**
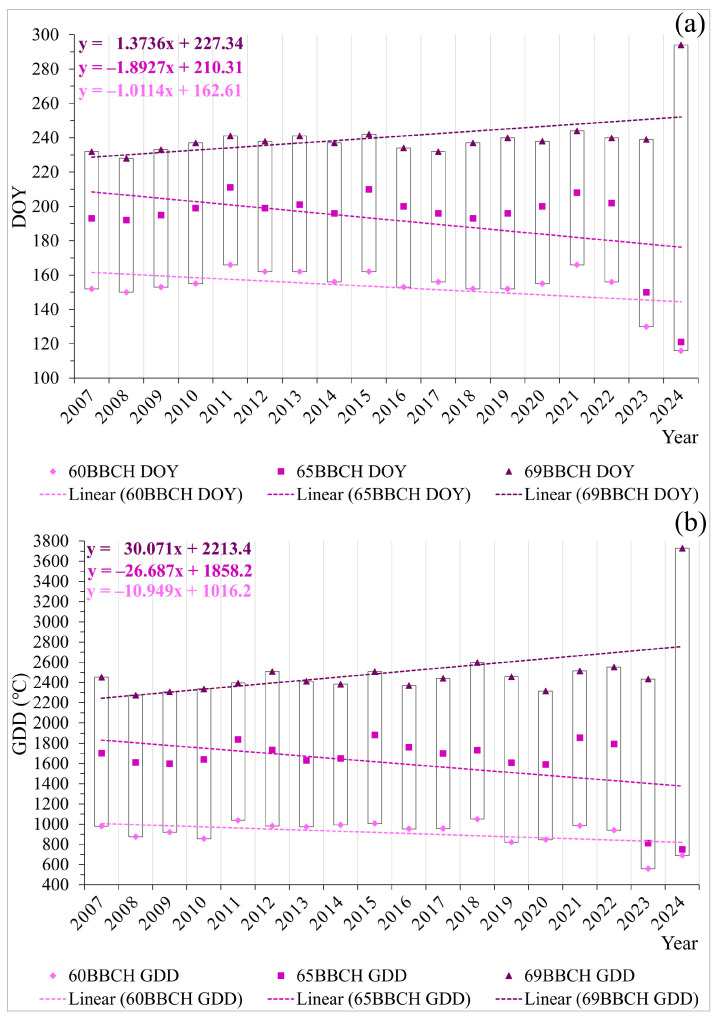
Display of (**a**) DOY and (**b**) GDD (°C) for the phenological patterns of primary flowering in *Rosa rugosa* Thunb. for the beginning of flowering (60BBCH), full flowering (65BBCH), and end of flowering (69BBCH), along with the corresponding linear trends, for the period 2007–2024.

For the recorded beginnings and full flowering, decreasing trends of DOY were observed, indicating that there are trends of earlier key phases of primary flowering (60BBCH and 65BBCH) over the 18 years of research (Figure 1a). The absolute difference between the earliest and latest 60BBCH is 50 days, but based on trend analyses, it was concluded that the flowering of *R. rugosa* has varied in accordance with changes in climatic parameters. The absolute difference between the earliest and latest 65BBCH is 90 days. The year 2024 stands out, with 60BBCH occurring on DOY 116 and 65BBCH on DOY 121, which is 37 and 71.3 days earlier compared to the average value of the previous analyzed years. For the last key phase of primary flowering, 69BBCH, an increasing trend was established (Figure 1a). The absolute difference between the earliest and latest 69BBCH is 66 days. The earliest end of flowering was on DOY 228 in 2008, which was 12.4 days earlier compared to the average value, while the latest was on DOY 294 in 2024, in the globally hottest year [25], which is 50.4 days later.

60BBCH was recorded after the accumulation of a heat sum of 559.6 °C (2023, DOY 130) to 1049.2 °C (2018, DOY 152). The mean accumulated heat value for 60BBCH, in the period from 2007 to 2024, is 912.2 °C. 65BBCH was recorded after the accumulation of a heat sum from 748.8 °C (2024, DOY 121) to 1883.3 °C (2015, DOY 210). The mean accumulated heat value for 65BBCH, in the period from 2007 to 2024, is 1604.7 °C. 69BBCH was recorded after the accumulation of a heat sum from 2273.3 °C (2008, DOY 228) to 3729.5 °C (2024, DOY 294). The mean accumulated heat value for 69BBCH, in the period from 2007 to 2024, is 2499.0 °C.

The current 2024 flowering started after an accumulation of 689.4 °C and ended (Figure 1b) after an accumulation of 3729.5 °C. The determined GDDs in the research period are variable. The linear trends for the three key phases of the phenological pattern of primary flowering (Figure 1a,b) confirm that the shift in DOY of these phenophases has no impact on the accumulation of heat, meaning that in the flowering pattern, they are recorded within specific ranges of accumulated heat (Figure 1b). A decrease in accumulated heat (GDD) for 60BBCH and 65BBCH and an increase for 69BBCH were observed, as confirmed by the linear trends, which are linked to extreme climatic events and the impact of other climatic parameters in the years of research.

The Mann–Kendall and Sen’s slope tests for the elements of the phenological pattern of primary flowering in *R. rugosa* over 18 years confirmed statistically significant differences for the end of flowering, specifically for 69BBCH DOY and 69BBCH GDDs (Table 4), where previously increasing trends were identified. However, the same tests for air temperatures and precipitation, from April to October when primary flowering occurred during the years of the study, did not confirm statistical significance between them for any of the analyzed seven months. Therefore, temperatures and precipitation were comparatively analyzed using the Spearman Rank coefficient, which confirmed only a strong positive correlation between air temperatures for the reference and study periods (0.975). From April to October, when primary flowering occurred during the study years, significant Spearman coefficients for the same parameters were observed in May (−0.553, moderate negative) and August (−0.483, weak negative), indicating that higher precipitation levels influenced a decrease in air temperature.

The values of the Spearman Rank correlation coefficient indicate a significant strong positive correlation between 60BBCH DOY and 65BBCH DOY (0.879), and a moderate positive correlation between 60BBCH DOY and 60BBCH GDD (0.637), 60BBCH DOY and 65BBCH GDD (0.694), as well as 65BBCH DOY and 65BBCH GDD (0.704). Additionally, there is a moderate positive correlation between 69BBCH DOY and 69BBCH GDD (0.551), as well as a strong positive correlation between 60BBCH GDD and 65BBCH GDD (0.791). Other correlations were not significant (Table 5).

Descriptive statistics (Table 6) were also used to assess the impact of climatic variables on the adaptability of *R. rugosa*. The standard deviation and other deviation parameters indicate a shift in the key events of the phenological pattern of primary flowering of *R. rugosa*.

**Figure 2:**
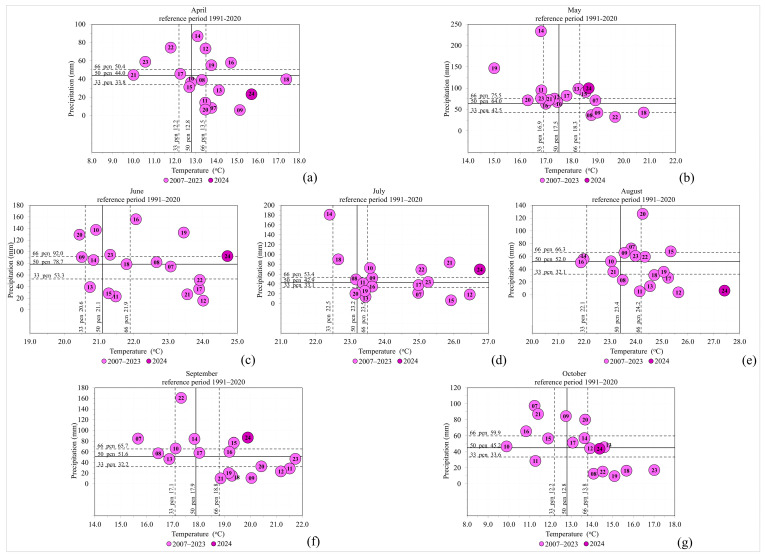
Average monthly air temperatures and total precipitation for the months (**a**) April, (**b**) May, (**c**) June, (**d**) July, (**e**) August, (**f**) September, and (**g**) October and their respective terciles for the period 1991–2024 (according to MMS Surčin data [25]).

Based on the previous analyses, it has been confirmed that the phenophase of primary flowering of *R. rugosa* was influenced by the interactions between years and climatic variables over the 18 consecutive years of research. Therefore, the average monthly air temperatures and precipitation amounts for the research period from 2007 to 2024, for April, May, June, July, August, September, and October, are shown in relation to each other in Figure 2, compared to the reference period of 1991–2020.

The earliest flowering onset (60BBCH) and full bloom (65BBCH) were recorded in 2024 (DOY: 116 and 121, respectively), when air temperatures in April were significantly above the upper tercile and precipitation was close to the lower tercile (Figure 2a), while in May, both air temperatures and precipitation were slightly above the upper tercile (Figure 2b). The latest 60BBCH was recorded in 2021 (DOY: 166), when air temperatures in April were significantly below the lower tercile, and precipitation was normal, while in May, temperatures were just above the lower tercile and precipitation was close to normal (Figure 2a,b). The latest 65BBCH was recorded in 2015 (DOY: 211), when air temperatures in June were slightly above normal and precipitation was significantly below the lower tercile (Figure 2c). The earliest end of flowering (69BBCH) was recorded in 2012 (DOY: 228), when air temperatures in August were significantly above the upper tercile, and precipitation was significantly below the lower tercile (Figure 2e), while the latest end of flowering was recorded in 2024 (DOY: 294), when air temperatures in June, July, and August were the farthest from the upper tercile during the period 1887–2024, and precipitation varied from the upper tercile (June and July) to significantly below the lower tercile in August (Figure 2c–e). During the period 2007–2024, the shortest flowering phase (76 days) occurred in 2010, when air temperatures in June were significantly below the lower tercile, slightly above the upper tercile in July, and at normal levels in August, while precipitation was significantly above the upper tercile (June), at the upper tercile (July), and at normal levels in August (Figure 2c–e). The longest flowering phase (179 days) was recorded in 2024, which, during the summer months, had air temperatures the farthest from the upper tercile compared to all previous years from 1991 to 2023 (Figure 2c–e). In the year with the longest flowering phase, temperatures and precipitation in September were significantly above the upper tercile, while in October, temperatures were above the upper tercile, and precipitation was normal (Figure 2f,g). The flowering phase of 2024 was preceded by an extremely warm winter—the warmest on record since measurements began at MMS Surčin in 1962, with a record-low number of frost days [25]. The shortest flowering phenophase (77 days) was recorded in 2017, which in the summer months is significantly above the upper tercile in terms of air temperatures and with precipitation below the lower tercile (Figure 2c–e). In the year of the shortest flowering phenophase in April, air temperatures were in the lower tercile, while in May, temperatures were in the upper tercile, and precipitation was normal in both spring months (Figure 2a,b). In the year with the longest flowering phenophase, the mean air temperature in the period 60BBCH-69BBCH was 22.3 °C, with the shortest 25.1 °C, which indicates that higher air temperatures shorten the length of flowering. The conclusion is confirmed by the mean negative correlation (Spearman coefficient: −0.53237), which indicates that the increase in air temperature moderately affects the shortening of the primary flowering period. The previously analyzed relationship between air temperature and precipitation, as well as the occurrence of extreme climatic events, is significant. Specifically, the phenological patterns of flowering were affected by variations in climatic parameters, which is in line with the statements of [26]. However, the effect of phylogeny, as the historical strategy of the species’ development, according to CaraDonna & Inouie [27], as well as the origin of *R.rugosa* and climatic and other characteristics of the range of the species, are also important. Over the course of all 18 years, the yield with abundance varied in accordance with the abundance of flowering.

In Table 6, statistical parameters are presented, indicating the variation in the number of days for the elements of the phenological pattern of primary flowering of *R. rugosa* and the average values of air temperatures during the relevant flowering periods. Notably, in 2024, 60BBCH started 39 days earlier compared to the average value for the period 2007–2023. 65BBCH in 2024 started 76 days earlier than the average value for the previous 17 years. 69BBCH ended 57 days later in 2024 than the average value for 2007–2023. The total primary flowering phase was longer by 96 days compared to the period 2007–2023. The shortest flowering phase occurred in the year when the average air temperature from 60BBCH to 65BBCH was approximately the same as the average air temperature for the entire primary flowering period. The longest flowering phase occurred in the year when the average air temperature for the primary flowering period was 22.3 °C, which is 1.3 °C lower than the average value for this parameter for the period 2007–2023, explaining the earlier start of primary flowering by two and a half months. It is noticeable that, in all years of the study, the period from 60BBCH to 65BBCH averaged 39.33 days, but in 2024, it was only 5 days long, which was influenced by the warm winter and spring in the globally warmest year [25]. The average number of days from 65BBCH to 69BBCH was 41.7 days in the period 2007–2023, while in 2024, it was 132.3 days longer. Given the pronounced variability of the phenological pattern elements of primary flowering during the study period, the average daily air temperatures during the relevant periods were determined, and descriptive statistics (Table 7) were used to assess the impact of air temperature on the primary flowering of *R. rugosa*.

The values of the Spearman Rank correlation coefficient indicate a significant, very strong positive correlation between the mean air temperature during the 60BBCH–65BBCH period and the mean air temperature over the entire primary flowering period (60BBCH–69BBCH), as well as a strong positive correlation between the 65BBCH–69BBCH period and the overall mean temperature during primary flowering (0.91973 and 0.79244, respectively). In other words, higher mean daily air temperatures between key phenological stages correspond to a higher overall mean temperature during the primary flowering period. A moderate positive correlation (0.59545) was also found between the mean air temperature in the 60BBCH–65BBCH period and that in the 65BBCH–69BBCH period. Furthermore, a moderate positive correlation (0.66894) between the length of the flowering phenophase and the number of days from 65BBCH to 69BBCH implies that a longer duration from full flowering to the end of flowering extends the overall length of the primary flowering phase. On the other hand, a moderate negative correlation (−0.69645) was recorded between the number of days from 65BBCH to 69BBCH and the number of days from 60BBCH to 65BBCH, indicating that a longer period from full flowering to its end corresponds to a shorter period from the onset to full flowering. A moderate negative correlation (−0.51122) between the number of days from 65BBCH to 69BBCH and the mean air temperature in the 60BBCH–65BBCH period further confirms the influence of air temperature on the phenological patterns of primary flowering. The Mann–Kendall and Sen’s slope tests applied to the same elements of the primary flowering phenological pattern of *R. rugosa* over the 18-year period confirmed only a statistically significant trend in the extension of the overall duration of primary flowering (Table 8).

### 2.3. Evaluation of Secondary Flowering

**Figure 3:**
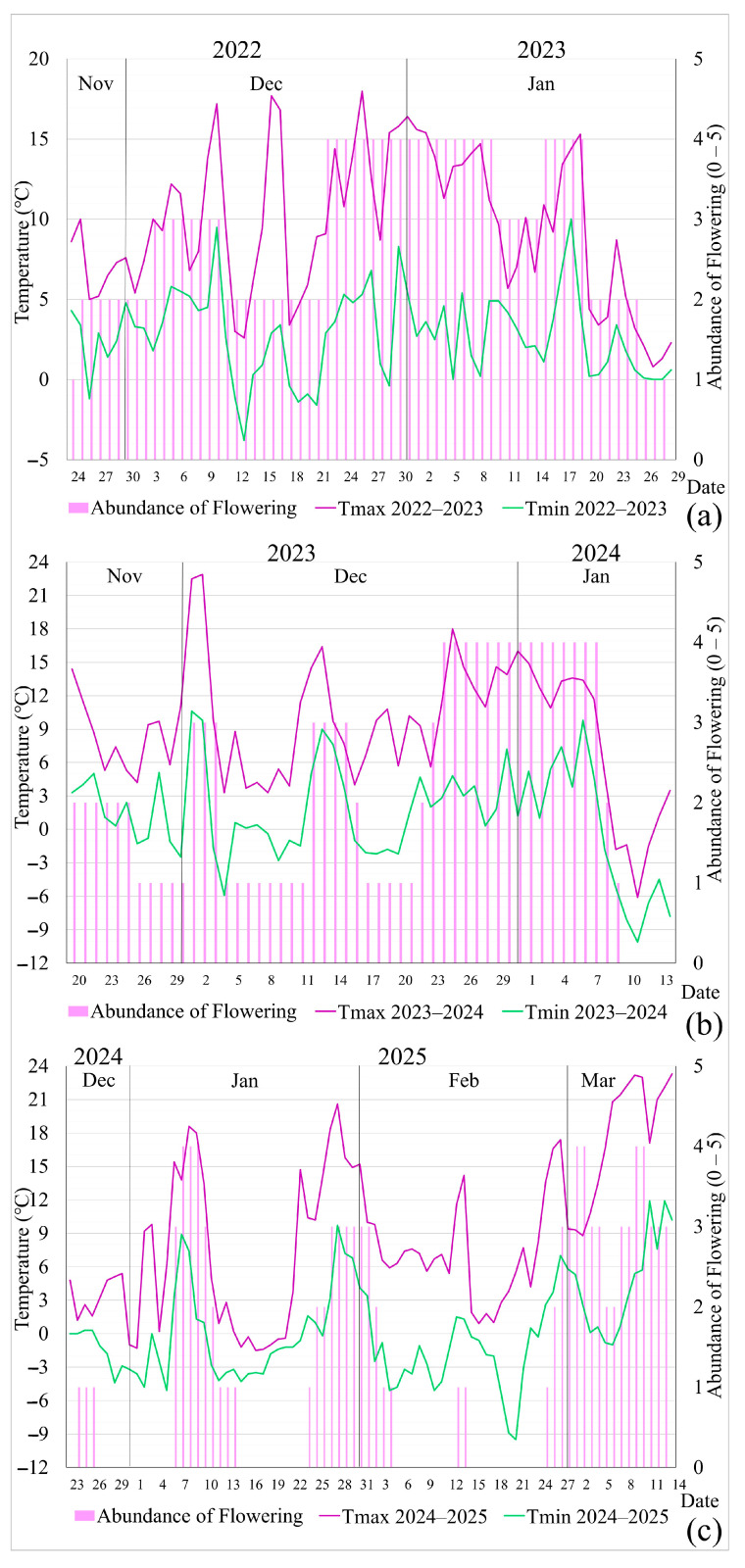
Graphical representation of the evaluation of secondary flowering abundance of *Rosa rugosa* Thunb. in Kupinovo (Vojvodina), based on original research and daily minimum and maximum air temperatures according to MMS Surčin data for (**a**) 2022–2023, (**b**) 2023–2024, and (**c**) 2024–2025.

**Figure 4:**
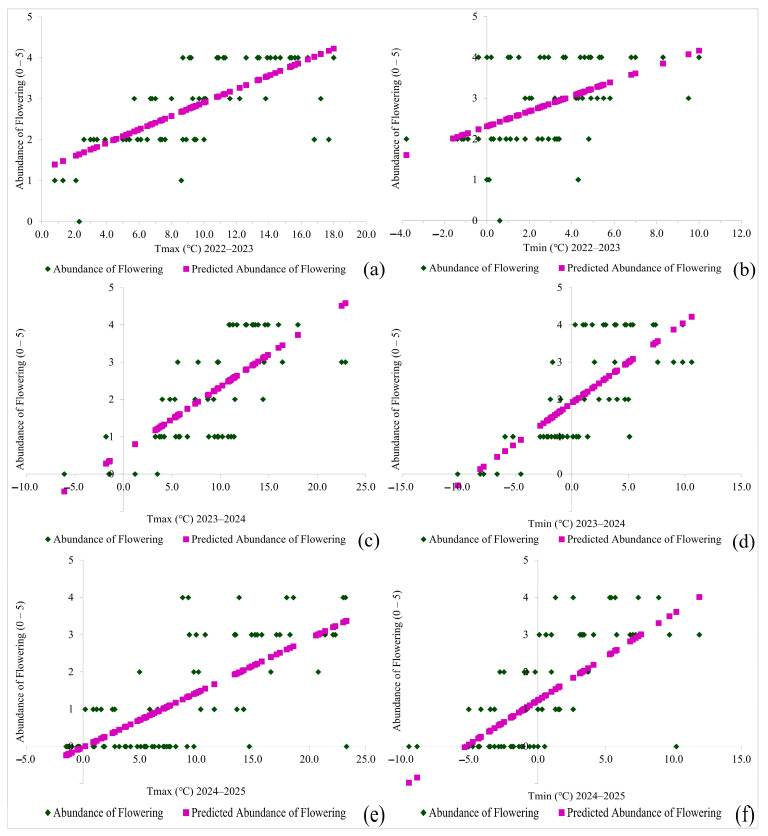
Scatter plots and predictions of the influence of daily maximum air temperature: (**a**) 2022–2023, (**c**) 2023–2024 and (**e**) 2024–2025, and minimum air temperature: (**b**) 2022–2023, (**d**) 2023–2024 and (**f**) 2024–2025 on the abundance of secondary flowering of *Rosa rugosa* Thunb. in Kupinovo.

The overall average score for the abundance of secondary flowering over the last three consecutive years was 2, indicating sparse flowering. The average abundance scores ranged from very sparse—score 1 (2024–2025), through sparse—score 2 (2023–2024), to moderate—score 3 (2022–2023). In all years of the study, abundance scores ranged from 0 to 4 (Figure 3a–c). Comparative analysis of the data identified the range of daily maximum and minimum temperatures associated with secondary flowering in *R. rugosa*: (a) for abundant flowering, the maximum was 13.6 °C and the minimum was 4.9 °C; (b) for moderate flowering, the maximum was 9.0 °C and the minimum was 4.2 °C. Spearman correlation coefficients, with a significance level of *p* < 0.05, confirmed the following: for 2022–2023, moderate positive correlations were found between maximum temperatures and flowering abundance (0.73566), between daily maximum and minimum air temperatures (0.5884), and between minimum temperatures and flowering abundance (0.50464); for 2023–2024, a strong positive correlation was observed between maximum temperatures and flowering abundance (0.761), as well as moderate positive correlations between minimum temperatures and flowering abundance (0.74791), and between daily maximum and minimum temperatures (0.72848); and for 2024–2025, moderate positive correlations were found between minimum temperatures and flowering abundance (0.74305), between maximum and minimum air temperatures (0.70888), and between maximum temperatures and flowering abundance (0.70337). Thus, the increase in maximum daily air temperatures was accompanied by a rise in minimum daily temperatures, and the increase in both variables had a moderate positive effect on the abundance of flowering (Figure 3a–c). The daily lowest maximum temperature of 0.8 °C and the lowest minimum temperature of −3.8 °C in 2022–2023 enabled a moderate average abundance of secondary flowering. As these temperatures dropped in 2023–2024 to −6.1 °C (maximum) and −10.1 °C (minimum), the abundance decreased to sparse. Very sparse flowering abundance in 2024–2025 was triggered by a daily lowest maximum temperature of −1.5 °C and a lowest minimum of −9.5 °C, with sub-zero temperatures persisting continuously for 11 days. The average maximum air temperature was 8.9 °C and the average minimum was 0.2 °C in 2024–2025, significantly lower compared to 9.0 °C (max) and 1.2 °C (min) in 2023–2024, and 9.5 °C (max) and 2.8 °C (min) in 2022–2023. Frost days interrupted secondary flowering and caused damage to flowers, although without the shedding of open flowers or flower stalks. No frost damage was recorded on unopened flower buds. Therefore, regression analyses were conducted based on daily maximum and minimum air temperatures for the three secondary flowering periods to determine their influence on flowering abundance (Figure 4a–f).

A growing trend is evident for both variables (Figure 4a–f) after approximating the extracted data, that is, determining the regression line, which confirms that with the increase in daily maximum and minimum air temperatures, the abundance of secondary flowering also increases.

During the secondary flowering of *R. rugosa* in 2022–2023, no damage was recorded, which is explained by the maximum temperature values that were not lower than 0 °C, and the minimum temperatures were below 0 °C on 8 out of 67 days, which were not continuous. Damage was recorded at a minimum maximum temperature of −1.8 °C and a maximum minimum temperature of −8.1 °C during the secondary flowering in 2023–2024, and during 2024–2025 at a minimum maximum temperature of −1.2 °C and a maximum minimum temperature of −4.3 °C when there were more than 4 frost days in a row. According to RHMS, frost days are days with maximum daily air temperatures lower than 0 °C. Damage to open flowers and partially open buds was recorded at the mentioned temperatures. Closed buds, leaves, and branches were free of damage during the secondary flowering periods analyzed. The statistical significance of these conclusions, at the *p* < 0.0001 level, is confirmed by the results of the regression ANOVA analysis (Table 9).

The results of the regression analysis for flowering abundance show that air temperatures from late autumn to early spring during the period 2022 to 2025 were statistically significant components that influenced the number of flowers, and consequently, the abundance of flowering.

## 3. Discussion

The research area is similar to the natural habitat of *R. rugosa* in Eastern Asia [28], which is why the differences in the phenological patterns of primary flowering over 18 consecutive years of research are correlated with climatic parameters. The onset of the primary flowering phenophase and subsequent stages of the phenological pattern are correlated with temperature and precipitation [29], and the later onset of flowering in European countries, including Serbia, is a result of partial adaptation to insect pollination [30].

In the conditions of a moderately continental climate, characteristic of Kupinovo, the average DOY for the onset of primary flowering (60BBCH) in the period 2007–2024 was 153 (2 June), aligning with [31,32], who noted that flowering in northern Hokkaido and Great Britain occurs in June–July. However, compared to China, where flowering occurs in May–June [33], flowering in Kupinovo began 30 days later, until 2024, when it started 5 days earlier, specifically on DOY 116 (25 April). Statistical parameters indicate a significant deviation in DOY, which is directly correlated with climatic parameters and extreme climatic events [34]. The year 2024 is marked as the hottest year in Serbia across all major meteorological stations, including Surčin (RHMZ). It is characterized by the hottest winter, spring, summer, as well as the warmest February, March, June, July, and August, with record-breaking values for average, maximum, and minimum annual temperatures [25]. Therefore, the higher average air temperatures during the period from the onset (60BBCH) to full (65BBCH) flowering, as well as from full (65BBCH) to the end (69BBCH) of flowering, influenced the short period of only 5 days from the onset to full flowering, or the necessary heat sums (GDD) accumulated earlier, which determine the key events in the phenological pattern of primary flowering. It was also recorded that in the previous year (2024), the primary flowering period was 91 days longer compared to the average duration of primary flowering in the period 2007–2023 in Kupinovo (Vojvodina). Specifically, the end of flowering (69BBCH) occurred on DOY 294 (20 October), which is 81 days later compared to studies in northern Hokkaido and Great Britain [31,32], and 112 days later compared to China [33]. The longest primary flowering phase occurred in 2024, which also saw four heatwaves during June, July, and August, with daily maximum air temperatures mostly above 35 °C [25]. The findings are consistent with previous research indicating that *R. rugosa* is highly adaptive and tolerant to both low and high temperatures, as well as to drought [35]. Its resistance to sub-zero conditions has been demonstrated through phenological monitoring and chlorophyll fluorescence measurements [29], while earlier studies confirmed its ability to survive temperatures as low as −35 °C [36]. Our original long-term observations in Kupinovo (2007–2025) combined with RHMZ data [25] suggest that *R. rugosa* can adapt within a temperature range exceeding 70 °C, from −35 °C to over 35 °C. The occurrence of secondary flowering during the 2022–2025 period supports this adaptability, aligning in part with findings that repeated flowering may continue sporadically until the first autumn frost [31]. The determined values for the duration and abundance of secondary flowering are of key importance for ecosystem services as well as landscape design.

The comparison of the findings in this study with research by other authors, who do not mention all the important characteristics of *R. rugosa* by omitting the optimal temperatures in late autumn and winter for flowering abundance [35,37,38], confirms the significance of the continuous 19-year study in Kupinovo (Vojvodina). According to the authors’ findings, these are the first results, in a three-year continuity, for secondary flowering. Considering the intensification of climate change, *R. rugosa*, a species with hermaphroditic flowers without nectar but with abundant pollen production of a strong scent, is crucial for pollinators, whose activity is highest on the first day the flower opens [39], especially since it is known that the species is pollinated by bees and bumblebees in its natural habitats [40,41]. The significance of secondary flowering in late autumn and winter is directly related to preserving ecosystem services, which has gained relevance following the announcement by the Serbian Beekeeping Organization (SPOS) regarding the death of bee colonies in Serbia in 2025, primarily in Vojvodina, with a mortality rate of 90%, caused by poor winter bee nutrition [42]. During the phenological observations, the presence of pollinators, especially bees, was recorded during the period of secondary flowering (2022–2025), confirming the species’ contribution to ecosystem services, particularly considering the estimation of the pollination ecosystem service value in Vojvodina, originating from bees (both wild and honeybees) [43]. The same author mentions three species in Vojvodina, *Andrena curvana* Warncke, *Tetraloniella lyncea* Mocsáry, and *Nomada bluethgeni* Stoeckhert, which are on the IUCN Red List, out of a total of 706 bee species found in Serbia.

In this connection, the findings of Winfree et al. [44] state that pollinators are a key component of global biodiversity who are vital for the maintenance of plant communities. However, whether pollinator declines have driven the evolution of floral and flowering traits is still unknown given historical collections and records of plant flowering phenology [45], pollinator richness and abundance [46], and pollinator–plant interactions [47]. The contribution to the mentioned questions is the findings of an eighteen-year study of flowering phenology, under conditions of climate change, which confirmed that Rugosa Rose is a species of high adaptive potential, which was pointed out by Li [48] and Galal et al. [49], stating that the species is characterized by efficient absorption and transport of cadmium.

The importance of this research and its findings is also evident because part of the Kupinovo area, Obedska Bara, is listed as an IBA (Important Bird Area) due to its biodiversity richness, particularly its ornithofauna, and has been recognized as an area of exceptional importance for birds since 1977. It is also listed as a wetland area under the Ramsar Convention [21,50,51]. The significance of flowering phenology in the context of climate change confirms the preservation of plant diversity, which positively influences overall biodiversity, including various insect and wildlife species [52]. Specifically, in Obedska Bara, there are 50 mammal species, 13 amphibians or reptiles, 222 bird species, and over 300 species of insects, including the bee species listed on the IUCN Red List [53]. The presence and adaptability of *R. rugosa* support nature-based solutions defined by the International Union for Conservation of Nature (IUCN) as strategies for the protection, sustainable management, and restoration of natural ecosystems [52].

The findings, along with the references [13,54], indicate that such research is significant for the development and conservation of roses with greater potential for flowering duration and abundance. Additionally, the results of our study form the basis for the preservation of ecosystem services and the formulation of an effective plan for their management. Specifically, the study clarifies the phenology of *R. rugosa* flowering, allowing policymakers, planners, urbanists, and landscape architects to influence the sustainable use of ecosystem services and improve quality of life. The findings highlight the species as adaptive, ornamental, and crucial for the preservation of ecosystem services, the design of nature-based solutions, and for cultivation for commercial purposes to produce raw materials for natural cosmetic and food products [55].

## 4. Materials and Methods

### 4.1. Study Area

**Figure 5:**
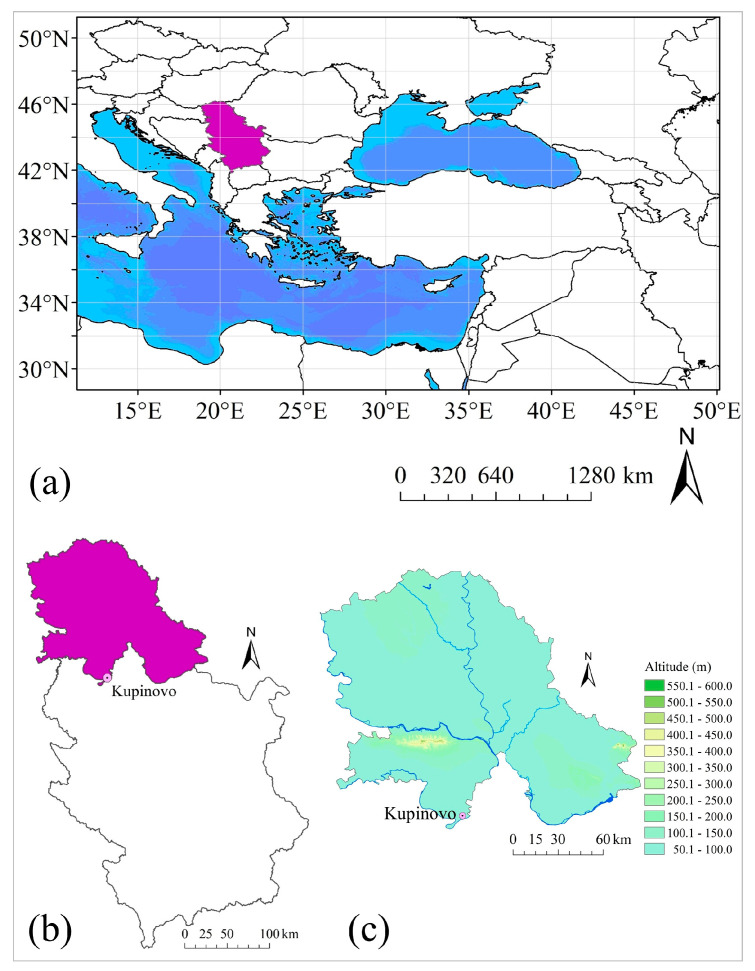
Study area: (**a**) position of Serbia (purple) in Europe, (**b**) Position of the Autonomous Province of Vojvodina (purple) and Kupinovo in Serbia, and (**c**) the research area on the topographic map of Vojvodina.

The research was conducted in Serbia, in the village of Kupinovo, located in the southern part of the Srem District and all of Vojvodina, on the left bank of the Sava River (Figure 5). Kupinovo is a cultural, historical, and tourist center of the southern part of the municipality and the only village in Srem that was once a royal city. Today, it is part of the municipality of Pećinci [56]. The village is home to the remains of the Kupinik Fortress (built at the end of the 13th or early 14th century as a military-border fortification for the Hungarian king), which is an immovable cultural monument of great significance. Kupinik is one of the best-preserved medieval fortification complexes in Vojvodina and is classified as one of the so-called “water cities” [57]. The oldest part of the village has been preserved and protected as an Ethno-village, and it contains some of the oldest houses in Vojvodina, which are legally protected as cultural heritage of Serbia [58].

Considering all of the above, during field reconnaissance conducted in 2006 for the establishment of a phenological monitoring network, *R. rugosa* was identified in Kupinovo and georeferenced at coordinates φ 44°42′39.09″ N and λ 20°03′37.52″ E, at an elevation of 75 m above sea level, situated on flat, non-exposed terrain, within an alluvial plain [59]. The soil type is classified as humogley, with a clay loam texture, neutral to slightly alkaline reaction, and high productive potential [60,61].

### 4.2. Climatic Data

The study utilized hourly and daily data of climate parameters from the Republic Hydrometeorological Service of Serbia (RHMZ) for the current reference period (1991–2020) and the period 2007–2025 [25], obtained from the main meteorological station in Surčin (φ44º47′54.44″ N, λ20º27′53.35″ E, elevation 99 m). The Surčin meteorological station was selected as the reference due to the similarity of environmental conditions and its proximity to the study area. For the reference period 1991–2020, climatological standard normals of the analyzed parameters were determined to enable the application of statistical climatological methods, including percentiles and corresponding terciles, for the study period 2007–2025.

### 4.3. Phenological Data

The data are the result of our own intensive phenological monitoring of flowering in the study area over a period of 19 consecutive years. Observations were carried out visually every other day throughout entire calendar years. The total number of phenological observations amounts to 3287, which were converted into day-of-year (DOY) values using specialized software. DOY coding assumes that 1 January corresponds to DOY 1, 2 January to DOY 2, and so on [62]. The DOY range is defined by the first and last dates of primary and secondary flowering. Secondary flowering was recorded at the end of the last three years (2022, 2023, and 2024), continuing into the spring of the following year (2023, 2024, and 2025). As a result, the DOY scale includes negative values (−) for key flowering events, with the lowest recorded DOY being −51 (2023). Namely, when primary or secondary flowering begins in the previous calendar year and ends in the following calendar year, the days preceding 1 January are given a minus sign (−). Day coding, in these cases, means that the date 31 December is equal to DOY − 1, 30 December = DOY − 2, 29 December = DOY − 3, etc., until −51 DOY, which is 10 November 2023 in this study.

In order to determine the impact of climate change on the ecosystem services of *R. rugosa*, the extended BBCH scale by [63] and the percentage of open flowers [64] were used to monitor the abundance of the secondary flowering phenophase during the period 2007–2025. These included the following: beginning of flowering—60BBCH (more than 10% of flowers open in the inflorescences), full flowering—65BBCH (more than 50% of flowers open in the inflorescences), and end of flowering—69BBCH (more than 80% of flowers in the inflorescences have faded). The abundance of flowering was assessed according to [65] on a scale where 0 indicates no flowers (0% of branches with flowers); 1—very weak (<20%); 2—weak (>20–<40%); 3—moderate (>40–<60%); 4—abundant (>60–<90%); and 5—maximum (>90%).

### 4.4. Processing of Data

Growing degree days (GDDs) were determined based on daily maximum (Tmax) and minimum (Tmin) air temperatures and the temperature threshold (Tt) using advanced formulas and corrective factors according to [24].

When Tmin > Tt, the following formula was used:

$$GDD=\frac{T_{max}+T_{min}}{2}-T_{t}$$

When Tmax < Tt, then GDD = 0.

When Tmax > Tt > Tmin, the following formula was applied:

GDD=εf(R)

where the coefficient is

$$\varepsilon =\frac{T_{max}-T_{min}}{2}$$

and

$$R=\frac{T_{t}-T_{min}}{T_{max}-T_{min}}$$

The value of the function f(R) was taken from the tables in [24] with respect to R.

Due to the recommendation for conditions of a moderately continental climate [34], the temperature threshold Tt = 5 °C was used. After forming the appropriate series of active temperatures, they were summed from 1 January for each day up to the DOYs for 60BBCH, 65BBCH, and 69BBCH for the first study year (2007). The procedure was repeated for each of the following 17 years, up to 2024 for primary flowering. By combining phenological and climatological data, the required heat quantities were determined, as phenophases do not occur on specific dates but after the accumulation of the appropriate heat sum [24]. The relationships between air temperatures and the abundance of secondary flowering were analyzed using correlations and linear regression.

The study utilized descriptive statistics, the Spearman Rank test (ρ), the non-parametric Mann–Kendall trend test, and the Sen’s slope test. The Mann–Kendall test was selected based on the recommendation of it being suitable for assessing trends in environmental data time series [34]. Sen’s slope is used in conjunction with the Mann–Kendall test to determine both the presence and magnitude of a trend. A positive Sen’s slope indicates an increasing trend, while a negative slope indicates a decreasing trend [66]. The Spearman Rank test indicates whether there is a consistently increasing or decreasing relationship between variables, with a probability of *p* < 0.05, and the strength and direction of the relationship are determined by the value and sign of the coefficient (−1 to 1) [67]. The strength of the correlation was interpreted as follows: 0 (no correlation), 0–0.24 (very weak), 0.25–0.49 (weak), 0.50–0.74 (moderate), 0.75–0.99 (strong to very strong), and 1 (perfect) [68].

Data processing was performed using the XLSTAT 2020, Past 4.11, and ArcGIS/ArcMap 10.8 software packages.

## 5. Conclusions

Based on our findings over 18 years of research (2007–2024), it is observed that the phenological patterns of primary flowering of *R. rugosa* in Kupinovo (Vojvodina) fluctuated, but overall, the temporal distribution showed a trend of earlier flowering and later cessation of flowering, as well as the occurrence of secondary flowering in late autumn and winter during the 2022–2025 period. The results of phenological observations indicate a possible shift in the timing of generative phenological phases in the event of an expected increase in the frequency of extreme warm periods. The start of flowering in 2024 was recorded 37 days earlier, and the end was later by 50.4 days compared to the 2007–2023 period. This phenological shift predictively suggests geographical movement and the potential for broader application. A comparative analysis of all variables confirmed that *R. rugosa* possesses the characteristics of a pioneer woody plant, demonstrating adaptability in flowering phenological patterns and yield that varied in accordance with the abundance of primary flowering from 2007 to 2024. It was also confirmed that the temperatures at the end of autumn and the beginning of spring during the 2022–2025 period were significant components influencing the abundance of secondary flowering.

The study confirms that *R. rugosa* is an adaptive and sustainable species in Kupinovo (Vojvodina) in the context of climate change, with no tendency for invasiveness. It is traditionally used for its ornamental and biomeliorative (soil erosion control) values. It is also significant due to its ecosystem services during the flowering period and fruiting, as the hypanthia serve as food for small birds and mammals, but it can also have economic value as a promising industrial plant. Phenological monitoring must continue in order to gain insights into the causes and consequences of changes in phenological patterns and questions related to the spatial and temporal organization of ecological communities and interactions. The conservation of biodiversity, alongside improving human well-being, is a significant challenge for Serbia and other developing countries. Additionally, further research should focus on locating *R. rugosa* in the ecosystems of Obedska Bara, which are interconnected into a unique ecological unit, a natural area exceptionally rich in species, with a mosaic of different biotopes ranging from open water bodies and wetlands to pastures, meadows, and high forest stands, alongside cultural and historical values as parts of the landscape.

## Figures and Tables

**Table 1:** Climatic variables for the reference period (1991–2020) and the study period 102 (2007–2024), the globally warmest year 2024, and its deviations compared to the reference period and the 2007–2023 period according to data from MMS Surčin.

**Mean air temperatures (°C)**													
**Months** **Period**	**1**	**2**	**3**	**4**	**5**	**6**	**7**	**8**	**9**	**10**	**11**	**12**	$\bar{x}$
$\bar{x}$ 1991/2020	1.0	3.0	7.5	12.9	17.6	21.4	23.2	23.2	18.0	12.8	7.4	2.2	12.5
$\bar{x}$ 2007/2023	2.0	4.2	8.1	13.3	17.8	22.1	24.2	24.0	18.8	13.2	8.3	3.4	13.3
2024	2.9	10.1	11.6	15.7	18.6	24.7	26.8	27.4	19.9	14.4	5.7	3.2	15.1
Deviation of 2024 from the norm 1991–2020	1.8	7.1	4.2	2.8	1.0	3.3	3.6	4.2	1.9	1.6	−1.7	1.1	2.6
Deviation of 2024 from the norm 2007–2023	0.9	5.9	3.6	2.4	0.9	2.6	2.6	3.4	1.1	1.2	−2.5	−0.2	1.8
**Mean maximum air temperatures (°C)**													
$\bar{x}$ 1991/2020	4.5	7.4	12.9	18.4	23.2	26.9	29.0	29.3	24.1	18.5	11.9	5.5	17.6
$\bar{x}$ 2007/2023	5.5	8.6	13.3	18.9	23.1	27.4	29.9	30.1	24.8	18.8	12.8	6.8	18.3
2024	7.1	15.5	17.2	21.6	23.7	29.9	32.2	33.6	25.9	20.3	10.4	5.9	20.3
Deviation of 2024 from the norm 1991–2020	2.5	8.1	4.4	3.1	0.6	3.0	3.2	4.3	1.8	1.8	−1.5	0.4	2.6
Deviation of 2024 from the norm 2007–2023	1.6	6.9	4.0	2.7	0.6	2.5	2.4	3.5	1.1	1.5	−2.4	−0.9	1.9
**Mean minimum air temperatures (°C)**													
$\bar{x}$ 1991/2020	−2.3	−1.0	2.6	7.1	11.8	15.4	16.8	16.9	12.6	7.9	3.6	−0.9	7.5
$\bar{x}$ 2007/2023	−1.4	0.1	3.2	7.3	12.1	16.2	17.7	17.5	13.2	8.4	4.4	0.3	8.2
2024	−0.8	5.2	6.3	9.3	13.6	18.4	20.1	19.8	14.1	9.3	1.4	0.7	9.8
Deviation of 2024 from the norm 1991–2020	1.5	6.2	3.7	2.2	1.8	3.0	3.3	2.9	1.6	1.3	−2.2	1.6	2.2
Deviation of 2024 from the norm 2007–2023	0.6	5.1	3.1	2.1	1.5	2.2	2.4	2.3	0.9	0.9	−3.0	0.4	1.5
**Sums and mean amounts of relative humidity (%)**													
Months Period	1	2	3	4	5	6	7	8	9	10	11	12	∑
$\bar{x}$ 1991/2020	85.0	78.0	68.8	64.9	66.2	66.4	63.1	62.5	68.8	74.6	79.9	85.3	72.0
$\bar{x}$ 2007/2023	82.9	75.1	66.1	61.6	65.4	64.5	58.6	58.9	65.1	72.2	78.4	82.8	69.3
2024	77.9	63.0	62.4	56.1	65.8	60.8	54.3	47.3	61.4	68.9	78.5	86.4	65.2
Deviation of 2024 from the norm 1991–2020	−7.1	−15.0	−6.3	−8.8	−0.4	−5.6	−8.8	−15.2	−7.4	−5.7	−1.5	1.1	−6.7
**Sums and mean amounts of precipitation (mm)**													
$\bar{x}$ 1991/2020	42.4	34.0	41.7	47.4	68.1	80.1	58.2	54.0	56.0	50.7	45.5	48.3	626.4
$\bar{x}$ 2007/2023	46.5	39.1	47.3	41.5	82.1	75.4	50.6	46.2	52.0	48.1	51.1	48.9	628.9
2024	37.1	4.8	27.7	23.3	99.8	92.5	69.4	6.5	86.8	44.0	45.6	63.2	600.7
Deviation (%) of 2024 from the norm 1991–2020	−12.5	−85.9	−33.6	−50.9	+46.5	+15.4	+19.2	−88.0	+55.0	−13.2	+0.2	+30.8	−4.1
Deviation (%) of 2024 from the norm 2007–2023	−20.2	−87.7	−41.5	−43.8	+21.5	+22.7	+37.2	−85.9	+66.9	−8.5	−10.8	+29.2	−4.5
**Number and mean number of days with precipitation ≥0.1 mm**													
$\bar{x}$ 1991/2020	12.4	11.0	10.5	11.6	13.0	11.6	9.6	8.0	9.3	10.0	10.2	12.7	129.9
$\bar{x}$ 2007/2023	14.5	12.2	11.2	11.9	14.1	11.2	8.4	7.9	9.5	9.9	10.9	13.5	135.3
2024	15	10	15	7	17	14	5	5	14	7	18	19	135
Deviation of 2024 from the norm 1991–2020	2.6	−1.0	4.5	−4.6	4.0	2.4	−4.6	−3.0	4.7	−3.0	−2.2	5.3	5.1
Deviation of 2024 from the norm 2007–2023	0.5	−2.2	3.8	−4.9	2.9	2.8	−3.4	−2.9	4.5	−2.9	−2.9	4.5	−0.3
**Sums and mean duration of sunshine (h)**													
$\bar{x}$ 1991/2020	78.6	106.9	163.0	200.0	240.7	272.0	298.7	281.3	206.4	166.1	102.3	67.0	2183.1
$\bar{x}$ 2007/2023	86.7	103.9	171.1	217.2	240.2	276.7	323.0	298.1	213.1	171.2	108.7	72.7	2282.6
2024	101.9	133.2	166.3	253.3	244.6	281.6	341.6	318.8	196.7	200.2	117.0	79.7	2434.9
Deviation of 2024 from the norm 1991–2020	23.3	26.3	3.3	53.3	3.9	9.6	42.9	37.5	−9.7	34.1	14.7	12.7	251.8
Deviation of 2024 from the norm 2007–2023	15.2	29.3	−4.8	36.1	4.4	4.9	18.6	20.7	−16.4	29.0	8.3	7.0	152.3
**Number and mean number of summer days (Tmax ≥ 25 °C)**													
$\bar{x}$ 1991/2020	0.0	0.0	0.2	2.5	12.0	20.1	25.7	26.2	13.7	4.1	0.1	0.0	104.7
$\bar{x}$ 2007/2023	0.0	0.0	0.2	2.8	11.4	21.0	27.8	27.0	15.8	4.8	0.1	0.0	110.9
2024	0	0	2	13	14	25	28	31	17	3	0	0	133
Deviation of 2024 from the norm 1991–2020	0.0	0.0	1.8	10.5	2.0	4.9	2.3	4.8	3.3	−1.1	−0.1	0.0	28.3
Deviation of 2024 from the norm 2007–2023	0.0	0.0	1.8	10.2	2.6	4.0	0.2	4.0	1.2	−1.8	−0.1	0.0	22.1
**Number and mean number of tropical days (Tmax ≥ 30 °C)**													
$\bar{x}$ 1991/2020	0.0	0.0	0.0	0.1	1.8	8.5	12.7	14.2	3.2	0.1	0.0	0.0	40.7
$\bar{x}$ 2007/2023	0.0	0.0	0.0	0.2	1.5	9.4	14.4	16.9	4.6	0.2	0.0	0.0	47.1
2024	0	0	0	1	0	15	22	28	10	0	0	0	76
Deviation of 2024 from the norm 1991–2020	0.0	0.0	0.0	0.9	−1.8	6.5	9.3	13.8	6.8	−0.1	0.0	0.0	35.3
Deviation of 2024 from the norm 2007–2023	0.0	0.0	0.0	0.8	−1.5	5.6	7.6	11.1	5.4	−0.2	0.0	0.0	28.9

**Table 2:** Mean monthly air temperatures and corresponding percentiles and terciles, along with their deviations for the period April–October 2024, based on data from the Surčin Meteorological Station, compared to the reference period.

Tmean (°C)	Perc. Cat. *	Tmean (°C) 1991–2020	1991–2020	1991–2020	1991–2020	Terciles **
	1991–2020		33.-Perc.	50.-Perc.	66.-Perc.	Cat.
April						
15.7	VW	12.9	12.2	12.8	13.5	1
May						
18.6	N	17.6	16.9	17.5	18.3	1
Jun						
24.7	EW	21.4	20.6	21.1	21.9	1
July						
26.8	EW	23.2	22.5	23.2	23.5	1
August						
27.4	EW	23.2	22.1	23.4	24.2	1
September						
19.9	W	18.0	17.1	17.9	18.8	1
October						
14.4	W	12.8	12.2	12.8	13.8	1

* Extremely warm (EW), very warm (VW), warm (W), normal (N), very cold (VC), extremely cold (EC). ** Warm (1), normal (0), cold (−1), categorization RHMZ.

**Table 3:** Monthly precipitation totals and corresponding percentiles and terciles, along with their deviations for the period April–October 2024, in the study area, compared to the reference period 1991–2020.

Sum (mm)	Perc. Cat. *	Sum (mm) 1991–2020	1991–2020	1991–2020	1991–2020	Terciles **
	1991–2020		33.-Perc.	50.-Perc.	66.-Perc.	Cat.
April						
23.3	D	47.4	33.8	44.0	50.4	−1
May						
99.8	W	68.1	42.5	64.0	75.5	1
Jun						
92.5	N	80.1	53.3	78.7	92.0	1
July						
69.4	W	58.2	33.1	42.9	53.4	1
August						
6.5	D	54.0	32.1	52.0	66.3	−1
September						
86.8	W	56.0	32.2	51.6	65.7	1
October						
44.0	N	50.7	33.6	45.2	59.9	0

* Extremely wet (EW), very wet (VW), wet (W), normal (N), dry (D), very dry (VD), extremely dry (ED). ** Wet (1), normal (0), dry (−1), categorization RHMZ.

**Table 4:** The results of Mann–Kendall and Sen’s slope tests for the phenological pattern elements of primary flowering of *Rosa rugosa* Thunb. in Kupinovo for the period 2007–2024, based on data from MMS Surčin.

Parameter\Test	Kendall’s Tau	*p*-Value *	Sen’s Slope **	Sen’s Slope Intercept
60BBCH DOY	−0.116	0.540	−0.167	490.833
65BBCH DOY	0.007	1.000	0.000	197.500
69BBCH DOY	0.442	0.013	0.533	−837.766
60BBCH GDD	−0.216	0.225	−5.288	11,615.088
65BBCH GDD	−0.098	0.596	−3.607	8928.316
69BBCH GDD	0.425	0.015	14.198	−26,187.576

* As the computed *p*-value is greater than the significance level 0.05, one cannot reject the null hypothesis H0. H0: There is no trend in the series. ** A positive Sen’s slope indicates an increasing trend, while a negative slope indicates a decreasing trend.

**Table 5:** Spearman’s correlation coefficients and *p*-value for DOY and GDD for *Rosa rugosa* Thunb., in the period 2007–2024, in the study area (significance level: *p*-value < 0.05).

Parameter	60BBCH DOY	65BBCH DOY	69BBCH DOY	60BBCH GDD	65BBCH GDD	69BBCH GDD
60BBCH DOY		0.056	0.154	**0.005**	**0.002**	0.823
65BBCH DOY	0.87944		0.070	0.052	**0.002**	0.974
69BBCH DOY	0.35092	0.43942		0.862	0.534	**0.019**
60BBCH GDD	**0.63756**	0.46815	0.04456		0.087	0.335
65BBCH GDD	**0.69373**	**0.7043**	0.15648	0.79154		0.197
69BBCH GDD	0.057204	0.0093217	**0.5513**	0.24045	0.31889	

Bold values are statistically significant (*p* < 0.05). Values in black are Spearman’s correlation coefficients; values in purple are *p*-value.

**Table 6:** Descriptive statistics for the investigated phenological parameters of primary flowering of *Rosa rugosa* Thunb. in Kupinovo, for 18 consecutive years (DOY determined based on own research, and GDD based on MMS Surčin data).

Parameters	60BBCH DOY	65BBCH DOY	69BBCH DOY	60BBCH GDD	65BBCH GDD	69BBCH GDD
Min	116	121	228	559.6	748.8	2273.3
Max	166	211	294	1049.2	1883.7	3729.5
Sum	2754	3462	4327	16,419.4	28,884.2	44,982.5
Mean	153	192.3333	240.3889	912.1889	1604.678	2499.028
Std. error	2.873135	5.17975	3.297988	29.37805	73.89117	75.31192
Variance	148.5882	482.942	195.781	15,535.25	98,278.28	102,093.9
Stand. dev	12.18968	21.9752	13.99218	124.6405	313.4937	319.5214
Median	155	197.5	238	954.75	1674.9	2437.3
25 prcntil	152	193	233.75	852.975	1606.45	2360.025
75 prcntil	162	201.25	241	986.85	1769.55	2511.2
Skewness	−2.05238	−2.6173	3.629506	−1.66862	−2.27627	3.709789
Kurtosis	4.85839	6.9570	14.53749	2.930922	4.612754	14.87591
Geom. mean	152.4873	190.8626	240.0456	902.6261	1563.187	2483.73
Coeff. var	7.96719	11.42596	5.820643	13.66389	19.53624	12.78583

**Table 7:** Descriptive statistics for the investigated phenological parameters and Tmean (mean air temperature) within the relevant elements of the phenological pattern of primary flowering of *Rosa rugosa* Thunb. in Kupinovo (2007–2024), based on original research and data from MMS Surčin [25].

Parameters	№ days 60BBCH–65BBCH	Tmean 60BBCH–65BBCH	№ Days 65BBCH–69BBCH	Tmean 60BBCH–65BBCH	№ Days 60BBCH–69BBCH	Tmean 60BBCH–69BBCH
N	18	18	18	18	18	18
Min	5	16.1	31	22.4	76	22.1
Max	48	26.3	174	25.8	179	25.9
Sum	708	401.8	883	434.5	1591	423
Mean	39.333	22.322	49.055	24.139	88.389	23.5
Std. error	2.473	0.602	7.925	0.228112	5.6152	0.258
Variance	110.118	6.526	1130.644	0.937	567.546	1.196
Stand. dev	10.494	2.555	33.625	0.968	23.823	1.094
Median	42	22.8	39	24.1	81.5	23.3
25 prcntil	39.75	21.375	37	23.4	79	22.6
75 prcntil	45	23.75	42.75	25.025	85.25	24.275
Skewness	−2.616	−1.078	3.472	−0.111	3.646	0.652
Kurtosis	7.057	1.802	12.574	−0.695	14.010	−0.346
Geom. mean	36.253	22.170	43.899	24.120	86.388	23.476
Coeff. var	26.679	11.445	68.545	4.009	26.953	4.655

**Table 8:** The results of the Mann–Kendall and Sen’s slope tests for elements of the phenological pattern of primary flowering of *Rosa rugosa* Thunb. in Kupinovo, for the period 2007–2024, based on original research and data from MMS Surčin.

Parameter\Test	Kendall’s Tau	*p*-Value *	Sen’s Slope **	Sen’s Slope Intercept
№ days 60BBCH–65BBCH	−0.027	0.909	0.000	42.000
Tmean 60BBCH–65BBCH	−0.124	0.495	−0.097	216.988
№ days 65BBCH–69BBCH	0.312	0.085	0.667	−631.000
Tmean 60BBCH–65BBCH	−0.124	0.495	−0.037	99.676
№ days 60BBCH–69BBCH	0.473	**0.008**	0.600	−723.600
Tmean 60BBCH–69BBCH	−0.059	0.762	−0.022	67.085

* The values in bold are slopes for statistically significant trends (*p* < 0.05). ** A positive Sen’s slope indicates an increasing trend, while a negative slope indicates a decreasing trend.

**Table 9:** ANOVA results for the influence of daily maximum and minimum air temperatures on the abundance of secondary flowering of *Rosa rugosa* Thunb., during the period 2022–2025, in Kupinovo (Vojvodina).

Parameter	df	SS	MS	F	Significance F
2022–2023					
Tmax					
Regression	1	38.1134	38.1134	73.431136	0.0000
Residual	65	33.73734	0.519036		
Total	66	71.85075			
Tmin					
Regression	1	16.44495	16.44495	19.292604	0.0000
Residual	65	55.40579	0.852397		
Total	66	71.85075			
2023–2024					
Tmax					
Regression	1	55.09212	55.09212	58.95538	0.0000
Residual	54	50.46146	0.934471		
Total	55	105.5536			
Tmin					
Regression	1	56.25659	56.25659	61.62356	0.0000
Residual	54	49.29698	0.912907		
Total	55	105.5536			
2024–2025					
Tmax					
Regression	1	89.75429	89.75429	88.8716	0.0000
Residual	80	80.79449	1.009931		
Total	81	170.5488			
Tmin					
Regression	1	87.56294	87.56294	84.41241	0.0000
Residual	80	82.98584	1.037323		
Total	81	170.5488			

## Data Availability

The original contributions presented in this study are included in the article. Further inquiries can be directed to the corresponding author.

## References

[1] Millennium Ecosystem Assessment (2005). Ecosystems and Human Well-Being: Desertification Synthesis.

[2] Scholte S., van Teeffelen A., Verburg P. (2016). Integrating socio-cultural perspectives into ecosystem service valuation: A review of concepts and methods. Ecol. Econ..

[3] Vujičić D., Vasiljević N., Radić B., Tutundžić A., Galečić N., Skočajić D., Ocokoljić M. (2024). Conceptualisation of the Regulatory Framework of Green Infrastructure for Urban Development: Identifying Barriers and Drivers. Land.

[4] IPBES Izveštaj o Globalnoj Proceni Satnja Biodiverziteta i Ekosistemskih Usluga. https://www.undp.org/sites/g/files/zskgke326/files/migration/rs/a84b97e3e718730ea61523ae36a97edcd1067336c966cb398a505abe7f15f909.pdf.

[5] Eldridge D.J., Bowker M.A., Maestre F.T., Roger E., Reynolds J.F., Whitford W.G. (2011). Impacts of shrub encroachment on ecosystem structure and functioning: Towards a global synthesis. Ecol. Lett..

[6] Moro M.J., Pugnaire F.I., Haase P., Puigdefábregas J. (1997). Mechanisms of interaction between a leguminous shrub understorey in a semi-arid environment. Ecography.

[7] Blaser W.J., Shanungu G.K., Edwards P.J., Olde Venterink H. (2014). Woody encroachment reduces nutrient limitation and promotes soil carbon sequestration. Ecol. Evol..

[8] House J.I., Archer S.R., Breshears D.D., Scholes R.J. (2003). Conundrums in mixed woody–herbaceous plant systems. J. Biogeogr..

[9] Harp D., Hammond G., Zlesak D.C., Church G., Chamblee M., George S. (2019). Flowering, drought and disease tolerance, and landscape performance of landscape roses grown under low-input conditions in north central Texas. HortTechnology.

[10] Ocokoljić M., Petrov D.J. (2022). Decorative Dendrology [Dekorativna dendrologija].

[11] Krüssmann G. (1974). Rosen Rosen Rosen Unser Wissen über die Rose.

[12] Smulders M.J.M., Arens P., Bourk P.M., Debener T., Linde M., Riek J., Leu L., Ruttink T., Baudino S., Saint-Oyant L.H. (2019). In the name of the rose: A roadmap for rose research in the genome era. Hortic. Res..

[13] Božanić Tanjga B., Ljubojević M., Đukić A., Vukosavljev M., Ilić O., Narandžić T. (2022). Selection of garden roses to improve the ecosystem services they provide. Horticulturae.

[14] (2019). Market Research Report. Rose Oil Market Size, Share and Trends Analysis Report by Application (Fragrance and Cosmetics, Pharmaceuticals, Food and Beverages), by Product (Organic, Conventional), and Segment Forecasts, 2019–2025.

[15] Popek R. (2002). Wild Roses of Poland—Róze Dziko Rosnące Polski.

[16] Lukasová V., Vido J., Škvareninová J., Bičárová S., Hlavatá H., Borsányi P., Škvarenina J. (2020). Autumn phenological response of European beech to summer drought and heat. Water.

[17] Ocokoljić M., Petrov D.J., Galečić N., Skočajić D., Košanin O., Simović I. (2023). Phenological Flowering Patterns of Woody Plants in the Function of Landscape Design: Case Study Belgrade. Land.

[18] Ocokoljić M., Petrov D.J., Galečić N., Skočajić D., Šišaković N., Simović I. (2024). The study of *Jasminum nudiflorum* Lindl. in urban green infrastructure in conditions of climate change in Belgrade, Serbia. Appl. Ecol. Environ. Res..

[19] Čukanović J., Ljubojević M., Djordjević S., Narandžić T., Petrov D.J., Ocokoljić M. (2024). The Impact of Climate Variability on the Blooming of *Fraxinus ornus* ‘Globosa’ as a Component of Novi Sad’s (Serbia) Green Infrastructure. Sustainability.

[20] Škvareninová J., Lukasová V., Borsányi P., Kvas A., Vido J., Štefková J. (2022). The effect of climate change on spring frosts and flowering of *Crataegus laevigata*—The indicator of the validity of the weather lore about “The Ice Saints”. Ecol. Indic..

[21] Cosmulescu S., Buican Stanciu A., Ionescu M. (2020). The influence of temperature on phenology of ornamental woody species in urban environment. Horticulture.

[22] Inoue T., Nagai S. (2015). Influence of temperature change on plant tourism in Japan: A case study of the flowering of *Lycoris radiata* (red spider lily). Jpn. J. Biometeorol..

[23] Petrov D.J., Ocokoljić M., Galečić N., Skočajić D., Simović I. (2024). Adaptability of *Prunus cerasifera* Ehrh. to Climate Changes in Multifunctional Landscape. Atmosphere.

[24] Lalić B., Ejcinger J., Dalamarta A., Orlandini S., Firanj Sremac A., Paher B. (2021). Meteorology and Climatology for Agronomists [Meteorologija i Klimatologija za Agronome].

[25] RHMZ. https://www.hidmet.gov.rs/ciril/meteorologija/klimatologija_godisnjaci.php.

[26] Silva I.A., Da Silva D.M., De Carvalho G.H., Batalha M.A. (2011). Reproductive phenology of Brazilian savannas and riparian forests: Environmental and phylogenetic issues. Ann. For. Sci..

[27] CaraDonna P.J., Inouye D.W. (2015). Phenological responses to climate change do not exhibit phylogenetic signal in a subalpine plant community. Ecology.

[28] eFlora “Flora of China” 2025. http://www.efloras.org/florataxon.aspx?flora_id=2&taxon_id=128746.

[29] Hakam N., Khanizadeh S., DeEll J.R., Richer C. (2000). Assessing Chilling Tolerance in Roses using Chlorophyll Fluorescence. HortScience.

[30] Bolmgren K., Eriksson O., Linder H.P. (2003). Contrasting flowering phenology and species richness in abiotically and biotically pollinated angiosperms. Evolution.

[31] Butkienė Z.P. (1971). Biological and biochemical characteristics of the rugosa rose. [Биологическая и биохимическая характеристика шиповника морщинистого]. Liet. TSR Moksl. Akad. Darb. Ser. C.

[32] Bruun H.H. (2005). Biological Flora of the British Isles. No. 239. Rosa rugosa Thunb. ex Murray. J. Ecol..

[33] Gu C., Robertson K.P. (2003). Rosa Linnaeus. Sp. Pl. 1: 491. 1753. Flora China.

[34] WMO (2021). State of the Global Climate 2021: WMO Provisional Report. World Meteorological Organization (WMO); 47. https://library.wmo.int/doc_num.php?explnum_id=10859.

[35] Baldzhieva M., Popova M. (1985). Some features of phenological development in Rosa rugosa Thunb. Nauchni Trudove, Vissh Selskostopanski Institut “Vasil Kolarov”.

[36] Jerzy M., Żyła S., Czekalski M. (1992). Róże 110 Odmian.

[37] Wlodarczyk Z., Ziernicka-Wojtaszek A., Kedizor R., Mazur J. (2023). Flowering phenology of shrub roses as a aensitive indicator of meteorological variability in Central Europe. J. Hortic. Res..

[38] Imperial Household Agency “Flower Calendar”. https://www.kunaicho.go.jp/e-event/hanadayori-cal.html.

[39] Dobson H.E.M. (1991). Pollen and flower fragrances in pollination. Acta Hortic..

[40] Fukuda H., Sakagami S.F., Yamauchi K., Matsumura T. (1973). Biofaunistic survey of wild bees in Hama-Koshimizu, Eastern Hokkaido. Jpn. J. Ecol..

[41] Kupianskaya A.N., Lelej A.S., Urbain B.K. (2000). The ants (Hymenoptera, Formicidae) of the Kuril Islands. Far East. Entomol..

[42] SPOS—Saveza Pčelarskih Organizacija Srbije. https://www.nin.rs/drustvo/vesti/73520/uginuce-pcela-u-srbiji.

[43] Markov Z. (2017). Fauna Insekata Polinatora u Vojvodini: Diverzitet, Brojnost i Procena Vrednosti Ekosistemske Usluge Polinacije. Doktorska disertacija.

[44] Winfree R., Bartomeus I., Cariveau D.P. (2011). Native pollinators in anthropogenic habitats. Annu. Rev. Ecol. Evol. Syst..

[45] Hart R., Salick J., Ranjitkar S., Xu J.C. (2014). Herbarium specimens show contrasting phenological responses to Himalayan climate. Proc. Natl. Acad. Sci. USA.

[46] Ollerton J., Erenler H., Edwards M., Crockett R. (2014). Extinctions of aculeate pollinators in Britain and the role of large-scale agricultural changes. Science.

[47] Burkle L.A., Marlin J.C., Knight T.M. (2013). Plant-pollinator interactions over 120 years: Loss of species, co-occurrence, and function. Science.

[48] Galal T.M., Majrashi A., Al-Yasi H.M., Farahat E.A., Eid E.M., Ali E.F. (2022). Taif’s Rose (*Rosa damascena* Mill Var. Trigentipetala) Wastes Are a Potential Candidate for Heavy Metals Remediation from Agricultural Soil. Agriculture.

[49] Li X. (2017). Study on Physiological and Accumulation Characteristics of Rosa Rugosa-Cv. to Copper, Cadmium and Lead Stress.

[50] Markićević M. (2022). Protection and improvement of SRP Obedska bara. [Zaštita i unapređenje SRP Obedska bara]. Zb. Rad. Geogr. Fak..

[51] Pravno Informacioni Sistem (2008). Uredba Vlade o Izmeni Uredbe o Zaštiti Specijalnog Rezervata Prirode “Obedska bara”. https://otvorenavlada.rs/uredba-obedska-bara0013-lat-doc/.

[52] Biswal B.K., Bolan N., Zhu Y.G., Balasubramanian R. (2022). Nature-based Systems (NbS) for mitigation of stormwater and air pollution in urban areas: A review. Resour. Conserv. Recycl..

[53] JP “Vojvodinašume” Plan Upravljanja SRP “Obedska bara” 2021–2030. https://www.vojvodinasume.rs/wp-content/uploads/2022/03/Plan-upravljanja-OB_2021-2030_medium-compressed.pdf.

[54] Tabaei-aghdaei S.R., and Rezaee M.B. (2003). Study of Flower Yield Variation in *Rosa damascena* L. Genotypes of Kashan. Iran. Rangel. For. Plant Breed. Genet. Res..

[55] Ma Y., Lin X., Liu R.F., Wu L.L., Li J.A. (2025). Analysis of Plant Growth and Flower Aromatic Composition in Chinese Rosa rugosa Cultivars Under Cadmium Stress. Horticulturae.

[56] Maraš Ž., Budovlačev Papić S., Nikolić Mirković M., Đuričić P., Pavlović M., Filipović D., Milutinović B., Vidaković I., Cvejić J., Dimovski K. (2006). Spatial Plan of the Municipality of Pećinci. [Prostorni Plan Opštine Pećinci].

[57] Bosnić D. (2022). Stari Gradovi Srbije.

[58] Pivac T. (2012). Vinski Turizam Vojvodine.

[59] Bukurov B. (1953). Geomorfološki prikaz Vojvodine. Zb. Matice Srp..

[60] Bogdanović Ž. (1982). Hidrološki Problem Srema. Ph.D. Thesis.

[61] Ivanišević P., Knežević M. (2008). Tipovi Šuma i Šumskog Zemljišta na Području Ravnog Srema. Monografija: “250 Godina Šumarstva Ravnog Srema”.

[62] Koch E., Bruns E., Chmielewski F.M., Defila C., Lipa W., Menzel A. Guidelines for Plant Phenological Observations. https://www.researchgate.net/publication/266211199.

[63] Meier U. (1997). BBCH-Monograph. Growth Stages of Plants. Entwicklungsstadien von Pflanzen. Estadios de las Plantas. Stades de Développement des Plantes.

[64] Buttler K.P., Schmid W. (1991). Anleitung für die Phänologischen Beobachter des Deutschen Wetterdienstes.

[65] Stilinović S. (1985). Seed Production of Forest and Ornamental Trees and Shrubs. [Semenarstvo Šumskog i Ukrasnog Drveća i Žbunja].

[66] Hirsch R., Slack J. (1984). Non-Parametric Trend Test for Seasonal Data with Serial Dependence. Water Resour. Res..

[67] Gilbert R.O. (1987). Statistical Methods for Environmental Pollution Monitoring.

[68] Horvat J., Mijoč J. (2012). Osnove Statistike.

